# Screening Biomarkers and Constructing a Predictive Model for Symptomatic Urinary Tract Infection and Asymptomatic Bacteriuria in Patients Undergoing Cutaneous Ureterostomy: A Metagenomic Next-Generation Sequencing Study

**DOI:** 10.1155/2022/7056517

**Published:** 2022-04-28

**Authors:** Qian Yuan, Rong Huang, Liping Tang, Lijuan Yuan, Li Gao, Yang Liu, Ying Cao

**Affiliations:** ^1^Department of Nursing, The First Affiliated Hospital of Nanchang University, Nanchang 330006, China; ^2^Nursing School, Nanchang University, Nanchang 330006, China; ^3^Genskey Medical Technology Co. Ltd., Beijing 102206, China; ^4^Department of Clinical Microbiology, The First Affiliated Hospital of Nanchang University, Nanchang 330006, China

## Abstract

**Objectives:**

To investigate the clinical diagnostic value of differential flora as biomarkers in patients with symptomatic urinary tract infection (UTI) and asymptomatic bacteriuria (ASB) undergoing cutaneous ureterostomy based on metagenomic next-generation sequencing and construct predictive models to provide a scientific reference for clinical diagnosis and treatment. *Material and Methods*. According to standard procedures, samples were taken from each patient for routine tests (urine, ureteral stent, and skin swab around the stoma). Cytokine levels in the blood were also detected. Urinary microflora were measured by mNGS, and potential biomarkers for distinguishing UTI and ASB were identified by differential flora. Finally, we generated the predictive models for ASB and UTI using the Lasso method and cytokine levels.

**Results:**

Urine culture was performed for 50 patients with cutaneous ureterostomy; 44 of these patients developed bacteriuria. The incidence of symptomatic bacteriuria was 54.55%. Biomarker analysis showed that *Propionimicrobium lymphophilum*, *Staphylococcus haemolyticus*, *Stenotrophomonas maltophilia*, *Ralstonia insidiosa*, and *Aspergillus sydowii* all had good predictive performance and were combined in a single model. The predictive model exhibited good prediction performance (area under the curve (AUC) = 0.8729, sensitivity = 80%, specificity = 83.3%, and cutoff = 1.855). We also identified a significant negative correlation between the weight sum of the abundance for these five characteristic pathogens (Sum_weighted_Reads) and levels of the cytokine IL-6 and IL-1*β* (*P* < 0.05).

**Conclusion:**

mNGS had a higher positive detection rate for pathogens in urine samples. The selected differential bacteria can be used as biomarkers of ASB and UTI, and the prediction model has good predictive performance. Analysis also showed that the occurrence of symptoms was related to individual immunity. Combined with the Sum_weighted_Reads cutoff and cytokine levels (IL-6 and IL-1*β*) of differential flora, it was possible to judge the severity of symptoms in cutaneous ureterostomy patients with bacteriuria and provide new insights for the treatment and intervention of ASB and UTI.

## 1. Introduction

Bladder cancer, with an estimated 573,000 new cases and 213,000 deaths in 2020, is the tenth most diagnosed cancer worldwide and is more common in men than in women [1]. Radical cystectomy (RC) with urinary diversion (UD) is the gold standard for the treatment of muscle-invasive bladder cancer (MIBC) and high-risk nonmuscle invasive bladder cancer (NMIBC) [2]. Cutaneous ureterostomy (CU) is one of the effective ways of performing UD and has a wide range of indications, involves relatively simple surgery, is associated with faster postoperative recovery, and is a reliable choice for older and frail populations [3]. However, a previous study showed that patients undergoing RC had the highest 30-day readmission rate among all urological oncological diseases; 14% of these readmitted cases were due to infection [4].

The most common postoperative infection types in CU patients are urinary tract infection (UTI) and asymptomatic bacteriuria (ASB); the latter is relatively common among older patients [5]. The clinical presentation of ASB and UTI shows distinct variations. Indwelling ureteral stents have been associated with a high incidence of bacteriuria and the incidence of chronic indwelling stent bacterial colonization, and bacteriuria has been reported to be 100%; these factors complicate the diagnosis of ASB and symptomatic UTI in patients with CU [6]. Globally, infection is a huge problem and associated with a high recurrence rate; these infections can lead to bacteremia, acute, and chronic renal insufficiency and can even become life-threatening [7].

Currently, the diagnosis of bacteriuria is mainly based on urine culture; however, this is a nonspecific laboratory test [8, 9]. According to the traditional viewpoint, the urine of normal and healthy subjects is sterile; positive and negative urine culture tests are usually regarded as the basis to distinguish whether an infection exists or not. However, routine urine culture methods are associated with certain limitations [10]. The urinary tract of normal and healthy controls is not “sterile;” rather, it possesses a core urinary microbiome. Approximately two thirds of these core bacteria are difficult to detect by traditional culture methods [11]. The intravesical instillation of some *Escherichia coli* strains have even been shown to prevent recurrent bacterial interference infection [12]. Therefore, the existence of bacteriuria does not necessarily imply infection. The detection of bacteria in urine does not in itself distinguish between symptomatic UTI and ASB. At present, in the unique group of patients undergoing radical cystectomy and urinary diversion, there is no clear criteria with which to distinguish UTI from ASB [13]. Rather, differential diagnosis mainly depends on urinary tract symptoms; UTI without obvious symptoms may be diagnosed as ASB, a condition that is relatively common in the elderly, those with cognitive impairment, and patients in an immunosuppressive state [14]. Therefore, it is vital that we develop novel and specific biomarkers that can reliably distinguish UTI from ASB.

Over time, it has become evident that asymptomatic bacteriuria, although classified as severe in terms of bacterial load, can no longer be considered an infection. Rather, such conditions are more like colonization, or in some cases, a risk factor; therefore, treatment is often unnecessary and may even be harmful [15, 16]. ASB does not require treatment, except for pregnant women and those undergoing invasive genitourinary surgery. However, in clinical practice, antibiotics are used empirically to treat bacteriuria and patients with ASB are often treated with antibiotics [17, 18]. Inaccurate and inadequate treatment coverage may lead to the acquisition of multidrug-resistant organisms, increased health care costs, and reduced patient satisfaction. Being able to distinguish between ASB and UTI is very important if we are to avoid unnecessary treatment in patients with ASB. At present, there is no clinically useful biomarker for the differential diagnosis of ASB and UTI in patients with CU.

Metagenomic next-generation sequencing (mNGS), as a new form of methodology, is rapidly being translated to clinical laboratories to permit the analysis of microorganisms and host genetic material in patient samples and can also be used for the unbiased detection of pathogens. mNGS has been applied to the pathogenic detection of infectious diseases and even the etiological diagnosis of some infectious diseases [19]. With the rapid development of mNGS technology, it has not only been confirmed that the urinary tract of healthy subjects has its own structure of unique flora but can also provide more comprehensive data relating to the microenvironment of an individual patient's urinary tract than traditional culture methods. This allows us to locate the microorganisms that may cause symptoms in a more accurate manner and thus helps us to move towards a model of precision medicine [20]. The flora of human urine is often associated with the occurrence of symptoms [21]. Moreover, there is a relationship between urinary microflora and a therapeutic response to urinary tract infection [22, 23]. Some studies have indicated that the diversity index of the bladder microbiome may eventually become a useful biomarker to distinguish ASB colonization from impending symptomatic UTI [24].

In the present study, we investigated the distributions of pathogens and factors related to bacteriuria in patients with CU. We also used mNGS and bioinformatics technology to identify potential biomarkers in the differential urinary flora of patients with ASB and UTI. Next, we used this information to build a predictive model to provide a reference for the differentiation of ASB and UTI in patients with CU. Finally, the model was combined with the weight sum of the abundance for these five characteristic pathogens (Sum_weighted_Reads) and cytokine levels to provide a scientific basis for the prevention of bacteriuria and the administration of antibiotics.

## 2. Materials and Methods

### 2.1. Patient Selection

All patients who were diagnosed with bladder cancer according to the bladder cancer diagnosis and treatment guidelines of the National Comprehensive Cancer Network (NCCN) [25] and underwent CU between December 1st, 2020 and January 31st, 2021 were selected as subjects for this study. We excluded patients who had been using steroids and immunosuppressants over the long term or those with infections other than in the urinary system or those who had received antibiotic treatment within the previous 14 days. Fifty patients undergoing CU were selected and urine samples were collected for urinary culture and mNGS tests; samples were removed from analysis if they were suspected of being contaminated or if they contained too few sequences.

Patients undergoing CU with bacteriuria were included according to their urinary test results. The diagnostic criteria for bacteriuria is mainly based on a positive urine culture: a bacterial count of ≥10^5 colony-forming units (CFU)/mL [26, 27]. ASB is defined as the presence of at least one type of bacterial growth in urine with a bacterial count ≥ 10^5 CFU/mL irrespective of whether pyuria is present or not and if there are no symptoms or signs attributable to UTI according to the guidelines published by the Infectious Diseases Society of America (IDSA) [28]. The US Preventive Services Task Force (USPSTF) recommends that the diagnosis for ASB should be the presence of bacteria in the urine without any signs or symptoms of UTI [29]. The European Association of Urology (EAU) guidelines on urological infections define ASB as a midstream sample of urine showing bacterial growth ≥ 10^5  CFU/mL without urinary tract symptoms. Published reports define UTI by urinalysis, bacteriuria, and clinical symptoms (fever, flank pain, and changes in the color, character, and smell of the urine) [30, 31].

A total of 44 CU patients with bacteriuria were included in this study. The included patients were divided into two groups by considering the clinical characteristics of their infection: a symptomatic UTI group (bacteriuria with clinical symptoms) and an ASB group (isolation of a bacterial organism with a count of at least 10^5 CFU/mL in a urine specimen from a patient without UTI symptoms).

### 2.2. Sample Collection

Ureteral stents and skin swabs around the stoma were collected and cultured according to standard procedures [32]. Cytokine levels in the blood were also detected. All samples were analyzed immediately after collection. Samples for mNGS were frozen in a refrigerator at −80°C and sent out to Genskey for central analysis. This study was approved by the ethics committee of the First Affiliated Hospital of Nanchang University, and all patients participating in this study signed an informed consent form. The protocol used for data collection in this study is in line with the Declaration of Helsinki.

### 2.3. Urinary Testing

Urinalysis was carried out with a AX4030 automatic urine dry chemical analyzer (ARKRAY Company of Japan). Urinary bacterial count and white blood cell count were determined with a UF1000i urine tangible component analyzer (Sysmex Company, Japan); all operating procedures were performed in strict accordance with the manufacturer's instructions. For urine culture, 1 *μ*L of clean midstream urine was inoculated onto a Columbia blood plate and a McConkey medium plate (Oxoid Company, UK); isolated bacteria were counted after culture at 37°C for 48 hours.

### 2.4. The Detection of Cytokine Levels

In total, twelve cytokine levels (including IFN-*α*, IL-1*β*, IL-6, IFN-*γ*, IL-10, IL-12P70, IL-17, IL-2, IL-4, IL-5, IL-8, and TNF-*α*) of each sample were detected by flow immunofluorescence photoluminescence with multiple microspheres using a kit from Qingdao Raiscare Biotechnology Co. Ltd. and a BD FACSCalibur flow cytometer.

### 2.5. DNA Extraction, Library Preparation, and mNGS Sequencing

Urine samples (5 mL) were collected using sterile screw freezing tubes. Then, 800 *μ*L was absorbed from the liquefied sample, transferred to a centrifuge tube, and centrifuged at 13,600 g for 5 min. Next, the supernatant was discarded and the precipitate was used for extraction. DNA was extracted with a Genskey Micro DNA Kit (1901, Genskey, Tianjin) in accordance with the manufacturer's instructions. The total mass of the extracted DNA, as determined by a Qubit dsDNA HS Assay Kit, needed to exceed 5 ng.

DNA libraries were constructed by DNA enzyme digestion (200-300 bp), end repair, A-tailing, adapter ligation, and PCR amplification using an NGS library construction kit (2012B, Genskey, Tianjin). Prior to sequencing, we used an Agilent 2100 Biological Analyzer (Agilent Technologies, Santa Clara, USA) to determine the quality of the DNA libraries in conjunction with qPCR to test the adapters. The concentration of the constructed DNA library needed to exceed 1 Nmol/L.

Finally, all of the sample DNA libraries were mixed in the form of DNA nanospheres; these were created by adding single-stranded circular DNA in 2–3 quantitative sets. The DNA nanospheres were loaded onto a sequencing chip and sequenced by the MGISEQ-2000 sequencing platform (MGI, Shenzhen, China). In each sequencing run, environmental control samples were used to monitor microbial DNA signals and microbial DNA signals generated by the background during batch processing; different ID spike variants were used to monitor for contamination between samples.

### 2.6. Bioinformatic Analysis of mNGS Data

Raw reads were quality filtered by fastp (version 0.19.5) [33] and Komplexity version 0.3.6 [34]; in particular, we evaluated data for adapter contamination, low-quality reads, and low-complexity reads. Reads that mapped to the human reference assembly GRCh38 were removed with bowtie2 version 2.3.4.3 [35]. Next, reads were aligned to a microorganism database consisting of approximately 12,000 genomes with SNAP version 1.0beta.18 [36], as described previously [37]. The mapped reads were classified based on the NCBI taxonomy assignment of the reference genomes. After filtering false-positive organisms, we then determined the species or genus abundance with Perl scripts.

Based on genera profiles, we also calculated the within-sample (*α*) diversity using the Shannon index and the Simpson index to estimate the richness of samples by QIIME2 [38]. Principal component analysis (PCA) was performed using the FactoMineR package in R software and a heat map was generated by the pheatmap package in R software, SVG scripts, and GraphPad Prism 8.

### 2.7. Statistical Analysis

The Wilcoxon test was used to compare differences across subgroups. Data analysis was performed using GraphPad Prism 8 software and R software. *P* < 0.05 was considered statistically significant.

## 3. Results

### 3.1. Demographic Characteristics

In total, 44 subjects (age range: 56–82 years) were included in this study. Of these 44 patients with CU, 36 patients were males (81.82%) and 8 patients were females (18.18%). There were 20 patients with ASB and 24 patients with symptomatic UTI (Table 1 and Supplementary Table S1). There was no significant difference between the ASB and UTI groups with regard to the age and gender. Most of the patients (in both groups) were elderly. Differential analysis between the two groups showed that patients with bilateral CU was more likely to have symptoms of infection than patients with unilateral CU, although the difference was not very significant (*P* = 0.093). There were differences between the two groups in terms of inflammatory cytokines (IL-6, IL-1*β*, and IL-12P70); only the levels of IL-6 (*P* = 0.025) and IL-1*β* (*P* = 0.033) were significantly different. There were no significant differences between the ASB and UTI groups with regard to other cytokines (Table 1).

### 3.2. Distribution of Pathogenic Bacteria in Urinary Cultures

**Figure 1:**
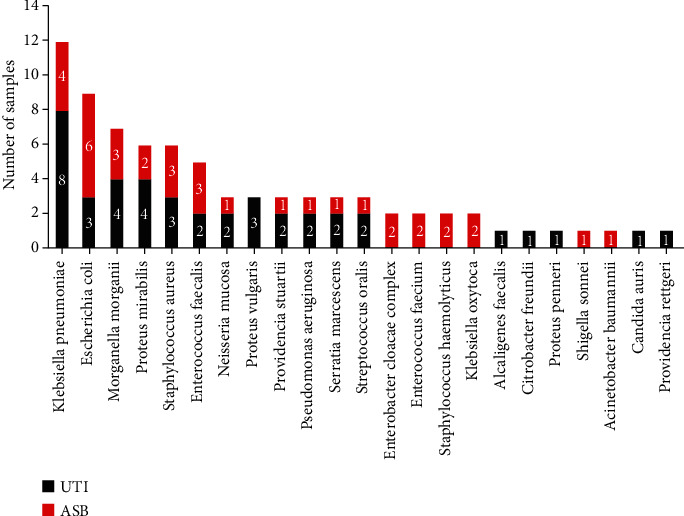
Urine culture pathogen detection spectrum and frequency.

Urinary analysis of samples from the 44 patients isolated 23 strains of pathogenic bacteria, most of which were bacteria (Figure 1 and Supplementary Table S1).

The main pathogens of infection in patients with a positive urine culture were Gram-negative bacilli: *Klebsiella pneumoniae* (ASB: 4 strains; UTI: 8 strains), *Escherichia coli* (ASB: 6 strains; UTI: 3 strains), *Morganella mortis* (ASB: 3 strains; UTI: 4 strains), *Proteus mirabilis* (ASB: 2 strains; UTI: 4 strains) and Gram-positive bacteria: *Staphylococcus aureus* (ASB: 3 strains; UTI: 3 strains), and *Enterococcus faecalis* (ASB: 3 strains; UTI: 2 strains). These six species of bacteria accounted for 57.69% (45/78) of the pathogens identified in urine cultures. These findings were consistent with the existing literature [39]. More than 2–4 different types of bacteria were cultured in the urine samples from 26 patients, including *Morganella* and mucinous *Neisseria*, *Klebsiella pneumoniae*, and *Escherichia coli*. Only one sample (S17) produced a fungus (*Candida auricularis*). *Klebsiella pneumoniae*, *Staphylococcus aureus, Proteus mirabilis*, and *Morganella* were detected more frequently in the UTI group than in the ASB group. The main pathogens (*Escherichia coli* and *Enterococcus faecalis*) were detected more frequently in the ASB group than in the UTI group. *Escherichia coli* accounted for 30% (6/20) of patients with ASB and 12.5% (3/24) of patients with symptomatic UTI.

### 3.3. Analysis of the Factors Affecting the Occurrence of Bacteriuria

**Figure 2:**
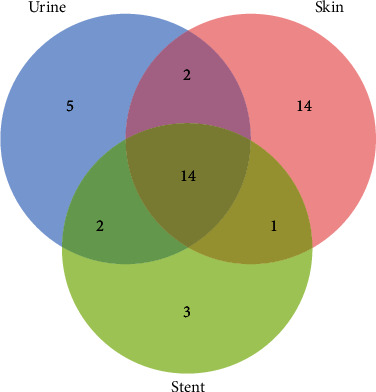
Ven diagram of cultured species of different kinds of samples. The 14 overlapping species of three groups (urine, skin, and stent) were *Candida auris*, *Enterococcus faecium*, *Enterobacter cloacae complex* sp., *Proteus vulgaris*, *Providencia stuartii*, *Proteus mirabilis*, *Morganella morganii*, *Enterococcus faecalis*, *Staphylococcus aureus*, *Escherichia coli*, *Klebsiella pneumoniae*, *Klebsiella oxytoca*, *Serratia marcescens*, and *Streptococcus oralis*.

In this study, while urine culture was carried out, skin swabs around the stoma and ureteral stents were also collected for bacterial culture. From the Ven diagram of the culture results of each sample, culture species of pathogen showed a decreasing trend from the skin to urine to ureteral stents. A total of 14 species are identical, including 6 pathogens frequently detected in the UTI group: *Klebsiella pneumoniae*, *Proteus mirabilis*, *Escherichia coli*, *Morgan*, *Staphylococcus aureus*, *and Enterococcus faecium* (Figure 2).

**Figure 3:**
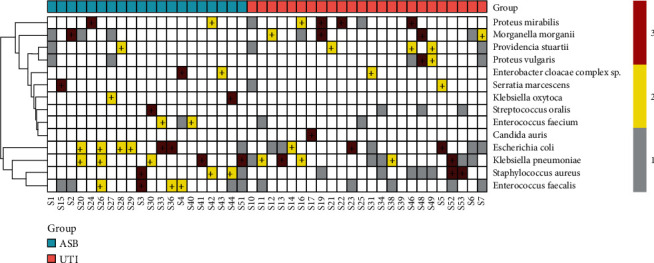
Heatmaps of detection frequencies for 14 species.

A heat map was made for the detection of these 14 bacteria in 44 patients (Figure 3). It can be seen from the chart that 63.6% (28/44) of patients could detect the same kind of bacteria from two or more kinds of samples at the same time and 47.7% (21/44) could detect the same kinds of bacteria from three kinds of samples at the same time. In addition, the culture results of all UTI and ASB groups were consistent with those of urine culture alone. The detection frequency of *Klebsiella pneumoniae*, *Staphylococcus aureus*, *Proteus mirabilis*, and *Morganella mortis* in the UTI group was higher than that in the ASB group. The first pathogen of infection in the ASB group was *Escherichia coli*, 35% (7/20) of the patients in the ASB group were cultured with *Escherichia coli*, and 33.33% (8/24) of the patients in the UTI group were cultured with *Escherichia coli*, which were higher than those cultured in urine alone. *Escherichia coli* can be cultured in stents or the skin in patients when *Escherichia coli* was cultured in urine (Supplementary Table S1). Therefore, it can be inferred that the pathogenic agents of infection in urine may come from the skin, stents, and other closely related external environments. Therefore, the longer stent placement time or numbers of stent placement were one risk factor affecting urinary tract infection, which is consistent with the results reported in other literature [40].

In addition, it has been reported that diabetes mellitus was a risk factor for urinary tract infection [30]. In this study, 3 patients (S18, S14, and S22) were complicated with diabetes, all of which were symptomatic urinary tract infections (Supplementary Table S1). It is suggested that diabetes may be one of the risk factors for the aggravation of bacteriuria in CU patients.

### 3.4. The Detection Performance of Urinary mNGS

Compared with the bacteria results from traditional urinary culture, the mNGS showed 97.44% consistency (Table 2). In total, 78 pathogens were identified by mNGS from urine samples; 76 of these samples were consistent with the results derived from urinary culture. Only two of the mixed infections were inconsistent between the two detection methods. The first case, S40 in the ASB group, was positive for *Enterococcus faecium* and *Staphylococcus haemolyticus* in the urinary cultures; mNGS did not detect *Staphylococcus haemolyticus*. In the second case, S31 in the UTI group, the urinary cultures were positive for *Klebsiella pneumoniae* and *Citrobacter freundii*; however, mNGS detected the sequence of *Citrobacter youngae*. mNGS was superior to urinary culture in terms of identifying bacterial species at a molecular level; therefore, the false-negative detection by mNGS in the second case may have been caused by incorrect identification at the species level in the urinary culture method. With regard to identifying bacteriuria, the positive rate of mNGS for ASB was the same as that for UTI; irrespective of whether cases were symptomatic or asymptomatic, the detection performance of mNGS was unaffected.

**Figure 4:**
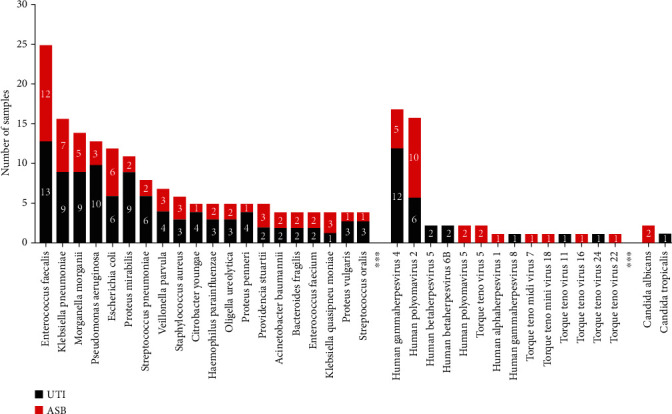
mNGS pathogen detection spectrum and frequency. Only twenty top species of bacteria by frequency were displayed.

The mNGS detection spectrum is known to be more extensive than traditional culture methods [41]. Pathogens with a higher abundance and frequency in the mNGS results were selected to create pathogen spectra (Figure 4). mNGS was able to detect bacteria (including *Enterococcus faecalis*, *Klebsiella pneumoniae*, *Morganella morganii*, *Pseudomonas aeruginosa*, *Escherichia coli*, *Proteus mirabilis*, *Streptococcus pneumoniae*, *Veillonella parvula*, *Staphylococcus aureus*, *and Citrobacter youngae*), fungi *(Candida albicans, Candida tropicalis*), and viruses (*Human gamma herpes virus 4* and *Human polyomavirus 2*).

### 3.5. Analysis of Microflora Diversity

**Figure 5:**
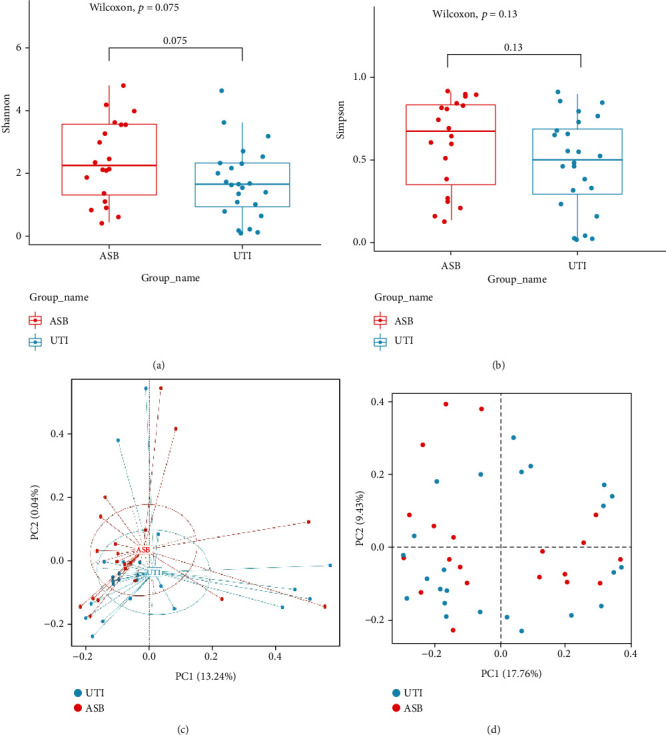
Metagenomic microbial diversity analysis. (a) Shannon *α* index diversity analysis. (b) *α* diversity Simpson index analysis. (c) Weighted PCoA using Bray-Curtis distance. (d) Unweighted PCoA.

Shannon and Simpson indices demonstrated that the diversity index for the ASB group was higher than that of the UTI group (Shannon: *P* = 0.075; Simpson: *P* = 0.13) (Figures 5(a) and 5(b)). The species diversity in the UTI group was lower than that in the ASB group, although there was no statistical significance, proving that the species structure was significantly similar when compared between the two groups of patients. Principal coordinate analysis (PCoA) further showed that there was a trend for separation between the UTI group and the ASB group (Figures 5(c) and 5(d)) and that the separation effect was better than that of unweighted PCoA, thus indicating that the effect of species abundance change was higher than that of species change. Therefore, the diversity of microflora was shown to decrease with the severity of infection in both the UTI and ASB groups. Furthermore, the species abundance could reflect the severity of infection. When the infection was serious, the abundance and diversity of the microflora changed; these results were consistent with the existing literature [42].

Sequencing reads from the 44 samples were greater than those from conventionally sequenced 20 million (Supplementary Table S2). All reads from the mNGS pathogens were normalized by equation (1).

(1) $$\begin{array}{c} {\text{Pathgeon}}_{\text{reads}}*\frac{20\text{ million}}{{\text{Total}}_{\text{reads}}}. \end{array}$$

Once the data had been normalized, we identified differences in the bacterial species between the UTI and ASB groups. Of all the pathogens detected by mNGS, 19 pathogens were significantly different when compared between the UTI and ASB groups (Table 3 and Supplemental Figure S1). Only the abundance of *Burkholderia cepacia* and *nematophilus* pathogenic bacteriashowed a significant increase in the symptomatic UTI group. The abundance of the 17 other bacterial species showed a significantly higher abundance in the ASB group, particularly Enterococcus faecalis, *Moraxella osloensis*, *Cutibacterium acnes*, *Stenotrophomonas maltophilia*, *Staphylococcus haemolyticus*, *Ralstonia insidiosa*, and *Propionimicrobium lymphophilum* (normalized abundance was >20 and the occurrence frequency was ≥50% in the ASB group). Of these, *Enterococcus faecalis* and *Stenotrophomonas maltophilia* were common opportunistic pathogens; *Moraxella osloensis* and *Cutibacterium acnes* were common colonization pathogens.

### 3.6. Generation of a Prediction Model for ASB and UTI

Next, the 19 species showing differential abundance between the two groups were investigated by Lasso regression; this led to the identification of five characteristic species and five characteristic weight coefficients (Table 4).

Then, we use these five characteristic species and weight coefficients to calculate the weight sum of the abundance for these five characteristic pathogens (Sum_weighted_Reads) and used this value as the final index to distinguish ASB from UTI. The formula for calculating the Sum_weighted_Reads is given in equation (2).

(2) $$\begin{array}{c} \text{Su}{\text{m}}_{\text{weighte}{\text{d}}_{\text{reads}}}=\overset{5}{\underset{1}{\sum }}\left(\frac{\text{Pi}\left(\text{reads}\right)}{\text{total reads}}*20\text{ million}\right)*{\text{weight}}_{\text{coefficient}}. \end{array}$$

In equation (2), P represents pathogen, i represents the serial number of the characteristics, and Weight_coefficient represents the weight coefficient of the characteristics in this model.

**Figure 6:**
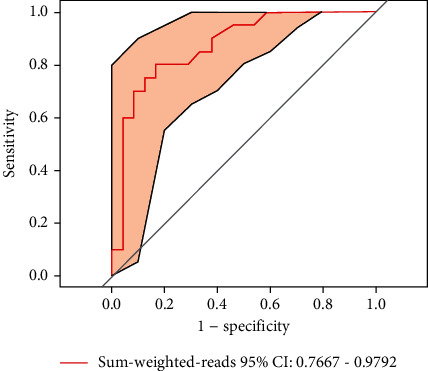
ROC curve of the prediction model.

Next, a ROC curve was generated to determine the accuracy (AUC = 0.8729; Figure 6); the sensitivity was 80%, the specificity was 83.3%, and the classification effect was good. According to a cutoff of 1.855 for this index, it follows that samples with an index < 1.855 are more likely to be classified as a UTI.

### 3.7. Correlation Analysis of Cytokine Levels

**Figure 7:**
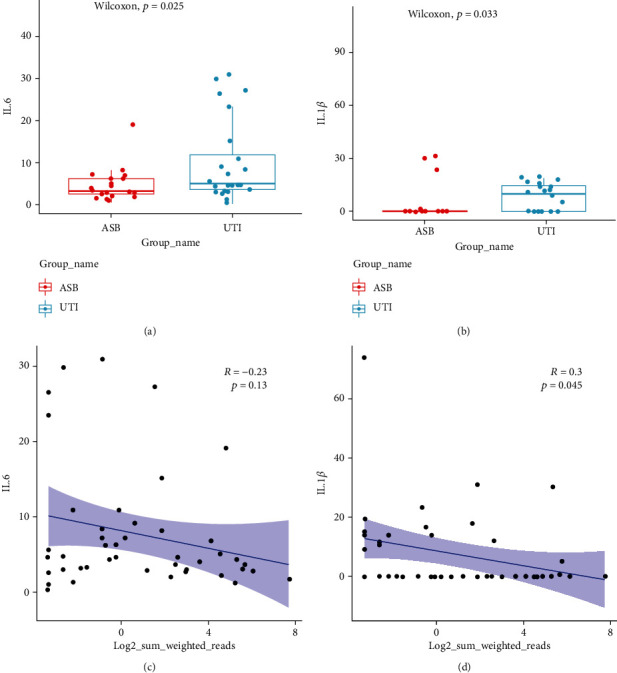
Association analysis of inflammatory factors. (a) Differential analysis of the inflammatory factor IL-6 between the ASB and UTI groups, (b) differential analysis of the inflammatory factor IL-1*β* between the ASB and UTI groups, (c) correlation analysis between Sum_weighted_Reads and IL-6 levels, and (d) correlation analysis between Sum_weighted_Reads and IL-1*β* levels.

We also performed correlation analysis on the levels of key clinical cytokines. Analysis showed that the levels of IL-6 and IL-1*β* in the UTI group were significantly higher than those in the ASB group; the increase in IL-6 was the most significant (Figures 7(a) and 7(b)).

IL-6 is a key mediator that plays an important role in inflammation and anti-inflammation during the process of wound repair. The expression levels of IL-6 can effectively reflect the severity of tissue and cell damage and can be used as the serological index for the clinical diagnosis of acute and chronic inflammation [43]. A sharp rise in the levels of IL-6 represents a red flag and has been widely recognized. IL-1*β* is an important inflammatory factor derived from the peripheral and central nervous systems [44].

The severity of infection is known to be associated with the levels of IL-6 and IL-8, at least to some extent. The levels of serum IL-6 and IL-1*β* in the acute stage of severe infection were previously shown to be significantly higher than those in the convalescent stage of severe infection and the acute stage of *Streptococcus pneumoniae* infection, while the abundance of *Streptococcus pneumoniae* in the acute stage was significantly higher than that in the convalescent stage (*P* < 0.05) [45].

Patients in the UTI group may have had immune dysfunction, thus leading to a significant increase in the serum levels of IL-1*β* and IL-6. Consequently, these levels can be used as reference indices to evaluate the severity of infection. The higher the levels of IL-1*β* and IL-6, the more serious the infection may be.

Next, we investigated the correlation between Sum_weighted_Reads and cytokine levels and found that Sum_weighted_Reads was negatively correlated with both IL-1*β* and IL-6 levels (*P* < 0.05). We found that this index exhibited a significant negative correlation with IL-1*β* levels; this was consistent with the reduction in abundance and species diversity in the UTI group (Figures 7(c) and 7(d)). These results suggested that we can combine the cutoff for the Sum_weighted_Reads for IL-1*β* and IL-6 to predict the severity of symptoms in patients with bacteriuria and thus identify appropriate forms of prophylactic medication in advance.

## 4. Discussion

This is the first study to use mNGS to identify biomarkers and construct a prediction model for UTI and ASB in patients undergoing CU. In this study, urine culture was performed in 50 patients with CU; of these, 44 patients developed bacteriuria. Consequently, there was a high incidence of bacteriuria in CU patients. The distribution of pathogenic bacteria in urinary cultures showed that the main pathogen associated with bacteriuria was Gram-negative bacilli, followed by Gram-positive cocci; these findings concurred with the general etiological characteristics of UTI. Furthermore, 2–4 different types of bacteria were cultured in the urine samples from 26 patients. The main risk factors for bacteriuria are the time and number of indwelling ureteral stents and diabetes mellitus. mNGS had a higher positive detection rate for pathogens in urine samples. Shannon and Simpson index analysis showed that the diversity index for the ASB group was higher than that for the UTI group. Thus, the identified differential bacteria (*Propionimicrobium lymphophilum*, *Staphylococcus haemolyticus*, *Stenotrophomonas maltophilia*, *Ralstonia insidiosa*, and *Aspergillus sydowii*) can be used as biomarkers for ASB and UTI. The generated prediction model showed good predictive performance. Furthermore, it is possible to judge the severity of symptoms of bacteriuria by combining the model with the cutoff of Sum_weighted_Reads and cytokines (IL-1*β* and IL-6) for differential flora.

The total incidence of bacteriuria in CU patients was 88%; 54.55% were diagnosed with UTI and 45.45% were diagnosed with ASB. ASB is common in many populations, although the prevalence is highly variable [46]. The incidence of ASB is 3.6% to 19% in older men and 40% in renal transplant recipients during the first month after surgery but can be as high as 100% in patients with chronic indwelling catheters [47]. In view of the large differences in the incidence of UTI and ASB reported in the literature, it is very important to define UTI and ASB in a specific manner if we are to better describe the real incidence of UTI in patients [48–50]. Most UTIs are caused by the ascending pathway, and the first step in its pathogenesis is the colonization of urinary tract pathogens in the tissue around the urethra. Second, urological pathogens may enter the urethra, thus leading to the occurrence of UTI or ASB; some pathogens may even reach the kidneys through the ureters [51]. In this study, the incidence of bacteriuria in CU patients was very high. After radical cystectomy and CU, the inevitable changes in the physiological structure can interfere with the normal barriers of the urinary system; this means that urinary reflux is common [52]. The implantation of ureteral stents can increase the risk of bacteriuria and bacterial colonization [53]. Therefore, ir is necessary to improve our understanding and monitor bacteriuria in patients with CU. The early identification of ASB and UTI is the premise of effective treatment, although there is no objective laboratory diagnostic index for ASB and UTI in patients with CU. Therefore, there is an urgent need to identify reliable biomarkers for differential diagnosis.

In total, 23 strains of pathogenic bacteria were isolated in this study, most of which were bacteria; only one sample produced a fungus (*Candida auricularis*). *Klebsiella pneumoniae*, *Staphylococcus aureus*, *Proteus mirabilis*, and Morgan were detected more frequently in the UTI group than in the ASB group. The main pathogens detected in the ASB patients were *Escherichia coli* and *Enterococcus faecalis*. A previous retrospective analysis of 1,133 patients after RC showed that the most common pathogens associated with UTI were *Escherichia coli*, *Enterococcus faecalis*, and *Klebsiella pneumoniae*; these findings concurred with those of the present study [53]. Gram-negative bacteria are known to be dominant in the microbiology of infection after RC (65–91% of isolates) [49]. Lipopolysaccharide (LPS) is expressed by Gram-negative bacteria and may interact with host cells such as leukocytes in the urinary system and uroepithelial cells. Although bacteria and/or LPS are present in the urine of both ASB and UTI patients, they seem to cause inflammation in UTI but not in ASB [54]. Perturbation of the urinary microbiome may indicate the development of a UTI. These pathogens existed in both ASB and UTI patients; therefore, we speculated that these populations would change and cause UTI when immunity was low or when there was a disruption in the balance of flora. Only one sample produced a fungus; the underlying causes for this may include different patient characteristics and the use of perioperative antibiotics. More than 2–4 different types of bacteria were cultured in the urine of some patients with bacteriuria. Most of the pathogenic bacteria arise from the commensal human urinary tract bacteria. Pathogenicity arises due to an imbalance in the relative abundance of pathogenic bacteria. Therefore, we speculate that there is a certain relationship between the production of bacteriuria and changes in the urinary flora, although the specific mechanisms involved need to be investigated further.

The etiology of UTI is also affected by host-related factors, such as age, diabetes, or catheterization [55]. The difference in age and gender between the ASB group and UTI group in the present study was not significant. Most patients in both groups were elderly. Bacteriuria is common in elderly patients, and its incidence is known to increase with age. In this study, because the patients undergoing CU surgery were elderly and frail, there was a lack of controls for different age groups. The female gender is known to be the main risk factor for the occurrence of bacteriuria [56]. Due to the fact that bladder cancer is more prevalent in males and because our sample size was limited, the results showed that males had a higher risk of bacteriuria. Furthermore, urine culture, ureteral stents, and skin swabs around the stoma were also collected for routine culture tests and we need to consider that ureteral stents are usually inserted after CU in patients with bladder cancer to prevent complications such as ureteral stricture. Bilateral CU is more likely to be associated with infection symptoms than unilateral ureterostomy; patients with positive *E. coli* urinary cultures can grow *E. coli* in both stents or the skin. Therefore, we inferred that the pathogens in urine may arise from the skin, stents, and the closely related external environment and that the main risk factors for UTI are the placement time or number of stents. In a previous study, Kehinde et al. investigated a large consecutive case series of 250 patients and concluded that the duration of stenting over 30 days correlated strongly with stent and stent tip culture results [57]. Other studies have shown that when ureteral stents were inserted for more than 6 weeks, there was a sharp increase in the abundance of bacteria in the urine [58]. Therefore, ureteral stents should be replaced in good time. Furthermore, it is important to maintain stents on a daily basis. Previous research has demonstrated an association between diabetes and the risk of UTI after RC and urinary diversion; this is consistent with the fact that diabetes is one of the risk factors for UTI in the normal population [59, 60]. Thus, targeted intervention according to the related factors of bacteriuria can effectively prevent and reduce the occurrence of bacteriuria.

According to the traditional viewpoint, the detection of urinary microorganisms is mainly based on standard urine culture in a clinical microbiology laboratory. However, urinary culture has some limitations; for example, these tests only allow the detection of aerobic bacteria and the rapid growth of a limited number of bacteria, such as *E. coli*. Metagenomic analysis using next-generation sequencing can provide information relating to microbial populations and help identify undetected microorganisms [61]. Previous studies have shown that mNGS has higher levels of sensitivity than traditional culture for certain types of sample such as blood, cerebrospinal fluid, and bronchoalveolar lavage fluid [62, 63]. Only a few reports have compared this new technology with traditional urinary culture. In this study, compared with conventional urinary culture, mNGS showed a consistency of 97.44%; only one bacterial result in each of the two cultures of mixed bacteria was inconsistent. We believe that there are specific reasons for this inconsistency. For example, mNGS is superior to clinical culture for the identification of species at the molecular level; therefore, the false-negative detection by mNGS in this case may be due to errors in the identification of specific species by the culture method. With regard to the identification of bacteriuria, the positive rates of detection for mNGS and urine culture were the same. Irrespective of whether a case was symptomatic or asymptomatic, the detection performance of mNGS remained unaffected. The detection spectrum for mNGS is more extensive than culture methods; in addition to bacteria, mNGS can also detect fungi and viruses. However, due to the high cost of mNGS detection, it may be a long time before this technology is widely used in clinical practice. As the cost of sequencing gradually decreases, the application of mNGS for urinary microbes is expected to be further developed.

The species diversity index for the ASB group was higher than that for the UTI group, although there was no significant difference, thus proving that the species structures of ASB and UTI were similar. According to the guidelines published by the Infectious Diseases Society of America (IDSA), ASB does not require screening and treatment except for pregnant women and patients undergoing invasive genitourinary surgery; this is because the effect of treatment cannot be improved [29]. A previous study showed that the frequency of UTI in patients with ASB was increased, although it was not clear whether symptomatic UTI develops from ASB; this needs further investigation [47]. PCoA analysis showed that the effect of species abundance change was higher than that of species change. When comparing the UTI and ASB groups, we found that the diversity of microflora decreased with the severity of infection and that the species abundance could reflect the severity of infection. When the infection was serious, the abundance and diversity of microflora changed; these findings were consistent with the literature [43].

Among all of the pathogens detected by mNGS, 19 pathogens were significantly different when compared between the UTI and ASB groups. The abundance of *Burkholderia cepacia* and nematophilus pathogenic bacteria was significantly higher in the UTI group. These are important pathogens. The abundance of the other 17 types of differential bacteria was significantly higher in the ASB group. Of the seven species of bacteria with significantly increased abundance in the ASB group, *Enterococcus faecalis* and *Stenotrophomonas maltophilia* were the most common opportunistic pathogens, while *Moraxella osloensis* and *Cutibacterium acnes* were the most common colonization pathogens. When the body has low resistance or in cases where there has been unreasonable use of antibiotics, opportunistic pathogenic bacteria can break through the protective barrier of the body, thus resulting in infection [46]. ASB is one of the main risk factors for the development of UTI [64], although individual immunity is also involved. Therefore, we inferred that a reduction in the abundance of beneficial bacteria and an increase in the abundance of pathogenic bacteria may lead to the occurrence of UTI although this requires further investigation.

Biomarkers are usually used to monitor and diagnose the pathological status of diseases. At present, biomarkers play an important role in the differential diagnosis and prediction of disease progression [65]. Therefore, it is of great significance to identify biomarkers with high accuracy to distinguish ASB and UTI so that we can improve the effective use of antibiotics and the prediction of infection progression. With the rapid development of bioinformatics and sequencing technology, a series of biomarkers had emerged. Some studies have shown that Akt and CD9 in urinary exosomes can be used as biomarkers to distinguish UTI from ASB [14]. However, it is technically difficult to measure the number of urinary exosomes because of the presence of outer membrane vesicles (OMVs) whose size and density are similar to that of exosomes; furthermore, exosomes that express Akt may not always express CD9. Recent studies have shown that there is a certain correlation between microflora and the production of symptoms. Important microflora could be used as diagnostic markers to distinguish ASB from UTI. Compared with existing indices, this strategy us simple and allows efficient intervention. Based on the use of mNGS to identify differences in the microflora as potential biomarkers, our current data show that *Propionimicrobium lymphophilum*, *Staphylococcus haemolyticus*, *Stenotrophomonas maltophilia*, *Ralstonia insidiosa*, and *Aspergillus sydowii* all performed well.

At present, most of the predictive models for urinary tract infection are based on the retrospective analysis of clinical data and the construction of predictive models according to risk factors. A previous retrospective analysis identified the characteristics of pathogens associated with catheter-related UTI by reviewing and analyzing relevant data from patients, including demographic, clinical, and microbiological data; the authors then constructed a predictive model with a sensitivity of <40% and a specificity of >90%. The sensitivity of this model for clinical prediction was low; therefore, it is necessary to develop more refined and sensitive tools [66]. The accuracy (AUC) of the predictive model developed in the present study was 0.8729, the sensitivity was 80%, the specificity was 83.3%, and the classification effect was good. According to an index cutoff of 1.855, it follows that when the index is <1.855, the sample is more likely to be diagnosed as a UTI. According to our predictive model, ASB and UTI can be identified effectively. However, in the future, it is necessary to expand the sample size and optimize the prediction performance of this model.

As a family of cytokines, interleukin plays an important role in regulating the immune response of infection and mediates the production of proinflammatory and anti-inflammatory signals [67]. During the acute stage of infection, both IL-6 and IL-1*β* are known to be expressed [46]. IL-6 is secreted by white blood cells and urothelial cells; these not only cause an acute response; they also lead to specific cellular and humoral immune responses. Therefore, IL-6 is an important inflammatory regulatory cytokine that acts in both the acute response and chronic response phases [67]. IL-1*β* is an important inflammatory factor from both peripheral and central nervous sources and can induce the production and release of many inflammatory factors, such as IL-8 and macrophage inflammatory protein 1 [45].

In the present study, we found that the levels of IL-6 and IL-1*β* in the UTI group were significantly higher than those in the ASB group and that the increase in IL-6 levels was the most significant. Related studies have shown that IL-6 is a highly sensitive and specific biomarker for UTI [67]. IL-6 and IL-1*β* play a certain role in inflammatory stress caused by clinical infection and the immune regulation of patients and are known to possess positive clinical significance in the postoperative tissue trauma repair of bladder cancer [68]. Inflammation is related to the migration of white blood cells to damaged tissue, thus destroying or challenging inflammatory triggers. Inflammation is a natural defense mechanism of the body. The initial stage of acute inflammation is caused by infectious and allergic inducement; this has a beneficial effect to a certain extent although persistent acute inflammation will lead to chronic inflammation and eventually lead to tissue damage [69]. It can be inferred that the patients in our UTI group also had immune dysfunction and the serum levels of IL-1*β* and IL-6 were significantly increased. These factors can be used as reference indices to evaluate the severity of infection. The higher the IL-1*β* and IL-6 levels, the more serious the infection.

Finally, we investigated the correlation between differential flora Sum_weighted_Reads and cytokine levels and found that Sum_weighted_Reads was negatively correlated with both IL-1*β* and IL-6. We found that this index showed a significant negative correlation with IL-1*β*, which was consistent with the observed reduction in UTI abundance and species diversity. These results suggest that we can combine the cutoff, IL-1*β* and IL-6 levels, and the Sum_weighted_Reads for specific bacteria to predict the severity of symptoms in patients with bacteriuria and thus provide prophylactic medication in advance.

## 5. Conclusion

Bacteriuria is relatively common among CU patients and may lead to significant morbidity. There were differences in species diversity and abundance between ASB and UTI. There are many potential consequences of microflora changes which may be important for generation of symptoms. mNGS has a higher pathogen-positive detection rate in urine samples. The selected differential bacteria can be used as biomarkers of ASB and UTI, and the prediction model has good predictive performance. The occurrence of symptoms is related to individual immunity. Combined with the cutoff of differential flora Sum_weighted_Reads and cytokines IL-1*β* and IL-6, the severity of symptoms in CU patients with bacteriuria can be judged, which provides a new perspective for the treatment and intervention of ASB and UTI. In general, we think that the findings of this study are of great significance because they provide a new insight into the occurrence, development, diagnosis, and treatment of cutaneous ureterostomy urinary tract infection.

### 5.1. Study Limitations

We are aware that there are some limitations in the statistical analysis of 44 cases in this study: the relatively small number of samples and the failure to dynamically monitor the changes in the composition of microflora in different periods may underestimate the accuracy of microbial diversity and relative abundance in our findings and reduce the performance of the prediction model. In the future, it is necessary to expand the sample size, monitor the dynamic changes of flora, and explain the possible pathogenicity of these specific bacteria, in order to evaluate their potential as new biomarkers and optimize the performance of the prediction model.

## Figures and Tables

**Table 1:** Patients' baseline characteristics.

Patients characteristics	ASB (*n* = 20)	UTI (*n* = 24)	*P* value	Normal range
Gender			0.6339	
Male	17 (85.0%)	19 (79.2%)		
Female	3 (15.0%)	5 (20.8%)		
Age (years)	66.45 (56–78)	67.79 (57–82)	0.524	
Hypertension			0.615	
Yes	6 (30.0%)	12 (50.0%)		
No	14 (70.0%)	12 (50.0%)		
Diabetes			0.111	
Yes	0 (0.0%)	3 (12.5%)		
No	20 (100%)	21 (87.5%)		
Ureterostomy type			0.093	
Double ureterostomy	11 (55.0%)	19 (79.2%)		
Unilateral ureterostomy	9 (45.0%)	5 (20.8%)		
Ureteral type			0.228	
Double ureter	17 (85.0%)	23 (95.8%)		
Single ureter	3 (15.0%)	1 (4.2%)		
IFN-*α* (pg/mL)	1.81 (0.78–3.08)	1.69 (0.83–2.92)	0.487	0–8.5
0–8.5	20 (100%)	24 (100%)		
>8.5	0 (0)	0 (0)		
IL-1*β* (pg/mL)	10.51 (0–73.67)	4.26 (0–31.2)	0.033^∗^	0–12.4
0–12.4	17 (85.0%)	15 (62.5%)		
>12.4	3 (15.0%)	9 (37.5%)		
IL-6 (pg/mL)	10.17 (0.25–30.95)	4.64 (1.07–19.1)	0.025^∗^	0–5.4
0–5.4	14 (70.0%)	12 (50.0%)		
>5.4	6 (30.0%)	12 (50.0%)		
IFN-*γ* (pg/mL)	5.55 (2.51–8.71)	7.64 (0.44–21.08)	0.248	0–23.1
0–23.1	20 (100%)	24 (100%)		
>23.1	0 (0)	0 (0)		
IL-10 (pg/mL)	0.86 (0.3–2.44)	0.94 (0.32–4.23)	0.888	0–12.9
0–12.9	20 (100%)	24 (100%)		
>12.9	0 (0)	0 (0)		
IL-12P70 (pg/mL)	0.02 (0–0.3)	0.2 (0–2.1)	0.063^∗^	0–3.4
0–3.4	20 (100%)	24 (100%)		
>3.4	0 (0)	0 (0)		
IL-17 (pg/mL)	1.74 (0.92–3.73)	2.16 (0.97–14.62)	0.229	0–21.4
0–21.4	20 (100%)	24 (100%)		
>21.4	0 (0)	0 (0)		
IL-2 (pg/mL)	0.7 (0–1.99)	0.82 (0–2.99)	0.396	0–7.5
0–7.5	20 (100%)	24 (100%)		
>7.5	0 (0)	0 (0)		
IL-4 (pg/mL)	0.6 (0–0.83)	0.64 (0.51–1.34)	0.981	0–8.56
0–8.56	20 (100%)	24 (100%)		
>8.56	0 (0)	0 (0)		
IL-5 (pg/mL)	2.17 (0–4.83)	2.27 (0.54–6.91)	0.934	0–3.1
0–3.1	20 (100%)	24 (100%)		
>3.1	0 (0)	0 (0)		
IL-8 (pg/mL)	11.23 (0–80.96)	7.08 (0.64–35.41)	0.352	0–20.6
0–20.6	19 (95.0%)	21 (90.0%)		
>20.6	1 (5.0%)	3 (10.0%)		
TNF-*α* (pg/mL)	1.27 (0–3.65)	1.6 (0–7.36)	0.502	0–16.5
0–16.5	20 (100%)	24 (100%)		
>16.5	0 (0)	0 (0)		
Nitrite (NIT)	0.22 (0–1)	0.06 (0–1)	0.206	Negative (−)
Negative (−)	15 (75.0%)	14 (58.3%)		
Positive (+)	1 (5.0%)	4 (16.7%)		
NULL	4 (20.0%)	6 (25.0%)		
Leukocyte esterase (LE)	2.06 (0–4)	1.91 (0–3)	0.760	Negative (−)
Negative (−)	2 (10.0%)	1 (4.2%)		
Suspective (±)	1 (5.0%)	2 (8.3%)		
Positive (+)	13 (33.3%)	15 (62.5%)		
NULL	4 (20.0%)	6 (25.0%)		
Bacteria count (U_BACT) (number/*μ*L)	2,714.71 (26.6––22,218.4)	1724.76 (52.1–7822.6)	0.800	0–32.8
0–32.8	0 (0.0%)	1 (4.2%)		
>32.8	15 (75.0%)	15 (62.5%)		
NULL	5 (25.0%)	8 (33.3%)		
Red blood cell count (RBC) (number/*μ*L)	1,051.36 (0.1–9,840.5)	824.05 (4.1–5,596.7)	0.711	0.2–13.8
<0.2	0 (0.0%)	1 (4.2%)		
0.2–13.8	3 (15.0%)	4 (16.7%)		
>13.8	12 (60%)	11 (45.8%)		
NULL	5 (25.0%)	8 (33.3%)		
White blood cell count (WBC) (number/*μ*L)	1,276.02 (87.1–4,670.7)	3,610.14 (7.5–36,212.4)	0.626	0–7.1
0–7.1	0 (0.0%)	0 (0.0%)		
>7.1	15 (75.0%)	16 66.73%)		
NULL	5 (25.0%)	8 (33.3%)		

Data are presented as *n* (%) or means (range).

**Table 2:** Consistency comparison between urine culture and mNGS.

Group	UTI	ASB	ALL
Urine_culture	42	36	78
Group	41	35	76
Consistency rate	97.62%	97.22%	97.44%

**Table 3:** Statistics of 19 different species between ASB and UTI groups.

Different pathogens	ASB_mean	UTI_mean	*P* value	ASB_num (*n*%)	UTI_num (*n*%)
*Enterococcus faecalis* ^∗^	12404.9	3495.8	0.0400	19 (95%)	19 (79.2%)
*Moraxella osloensis* ^∗^	22.9	9.0	0.0371	17 (85%)	15 (62.5%)
*Burkholderia cenocepacia* ^∗^	0.0	0.6	0.0342	0 (0%)	5 (20.8%)
*Pandoraea norimbergensis*	2.5	2.2	0.0337	7 (35%)	2 (8.3%)
*Xenorhabdus nematophila* ^∗^	0.8	1.7	0.0309	1 (5%)	8 (33.3%)
*Cutibacterium acnes* ^∗^	122.3	58.4	0.0283	20 (100%)	24 (100%)
*Eikenella corrodens*	293.7	0.0	0.0248	4 (20%)	0 (0%)
*Neomicrococcus aestuarii*	1.1	0.0	0.0248	4 (20%)	0 (0%)
*Staphylococcus warneri*	1.4	0.0	0.0248	4 (20%)	0 (0%)
*Paraburkholderia fungorum*	3.8	1.0	0.0149	11 (55%)	5 (20.8%)
*Aspergillus sydowii*	0.8	0.2	0.0136	10 (50%)	4 (16.7%)
*Malassezia globosa*	4.2	0.8	0.0094	16 (80%)	14 (58.3%)
*Stenotrophomonas maltophilia* ^∗^	56.2	1.0	0.0064	14 (70%)	9 (37.5%)
*Legionella drozanskii*	0.5	0.0	0.0047	6 (30%)	0 (0%)
*Acidovorax* sp. KKS102	5.5	3.8	0.0033	15 (75%)	6 (25%)
*Staphylococcus haemolyticus* ^∗^	44.2	0.0	0.0020	7 (35%)	0 (0%)
*Desulfocarbo indianensis*	5.2	0.3	0.0018	9 (45%)	1 (4.2%)
*Ralstonia insidiosa* ^∗^	56.9	1.4	0.0002	14 (70%)	6 (25%)
*Propionimicrobium lymphophilum* ^∗^	807.6	0.0	0.0001	10 (50%)	0 (0%)

∗For pathogens with normalized abundance > 20 and occurrence frequency ≥ 50% in ASB, indicating higher abundance in UTI.

**Table 4:** Weight coefficients of five characteristics.

No.	Pathogens	Weight coefficient
1	Propionimicrobium_lymphophilum	0.016094976
2	Staphylococcus_haemolyticus	0.038285169
3	Stenotrophomonas_maltophilia	0.063515774
4	Ralstonia_insidiosa	0.170320578
5	Aspergillus_sydowii	0.462107080

## Data Availability

Sequencing data from the 50 samples (with human reads removed) are available in NCBI Sequence Read Archive (SRA) with the BioProject identifier PRJNA779226.

## Supplementary Materials

Supplementary Materials Supplementary Table S1: clinic characteristics of patients included in this study. Supplementary Table S2: data production of 44 samples.
