# Supplementary figures and images for: Screening Biomarkers and Constructing a Predictive Model for Symptomatic Urinary Tract Infection and Asymptomatic Bacteriuria in Patients Undergoing Cutaneous Ureterostomy: A Metagenomic Next-Generation Sequencing Study
Source: Dis Markers. 2022 Apr 28;2022:7056517. doi: 10.1155/2022/7056517 (PMC9072028; doi:10.1155/2022/7056517)

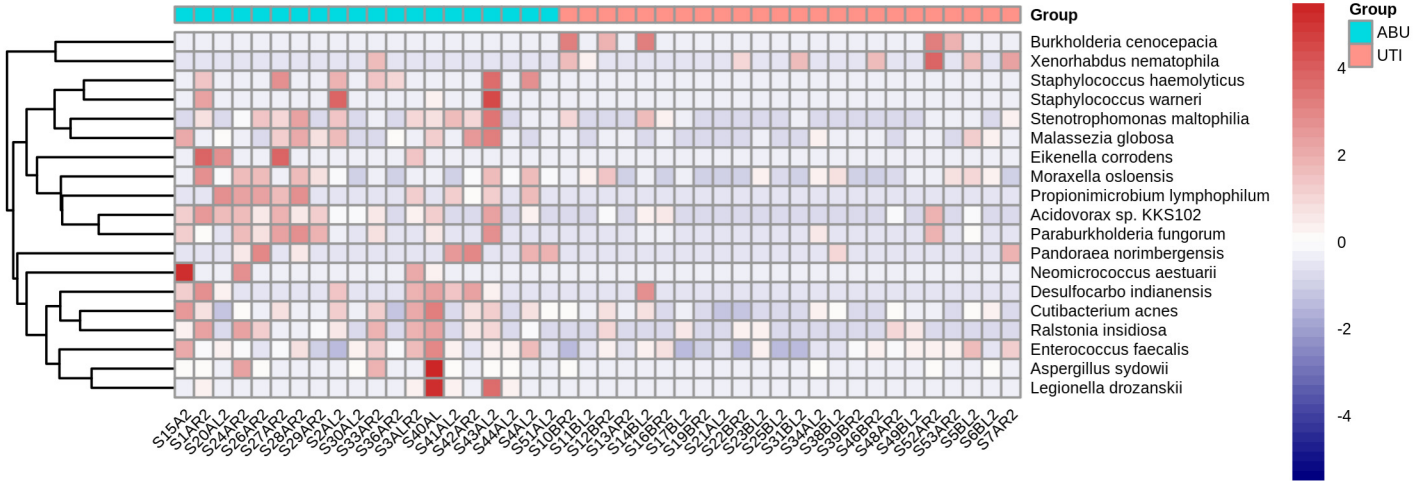

Supplement: Supplementary Materials — Supplementary Table S1: clinic characteristics of patients included in this study. Supplementary Table S2: data production of 44 samples. [file 7056517.f1.zip › Supplemental_Figure_S1.pdf]
