# Supplementary material for: Screening Biomarkers and Constructing a Predictive Model for Symptomatic Urinary Tract Infection and Asymptomatic Bacteriuria in Patients Undergoing Cutaneous Ureterostomy: A Metagenomic Next-Generation Sequencing Study
Source: Dis Markers. 2022 Apr 28;2022:7056517. doi: 10.1155/2022/7056517 (PMC9072028; doi:10.1155/2022/7056517)
Supplement: Supplementary Materials — Supplementary Table S1: clinic characteristics of patients included in this study. Supplementary Table S2: data production of 44 samples. [file 7056517.f1.zip › Supplementary Table S1_clinic characteristics of patients included in this study.doc]

| **Supplementary Table S1 clinic characteristics of patients included in this study** | | | | | | | | | | | | |
| --- | --- | --- | --- | --- | --- | --- | --- | --- | --- | --- | --- | --- |
| **NO** | **Sample** | **Group** | **Sex** | **Age** | **Chronic Underlying Diseases Type** | **Infection characteristics** | **Urine Culture** | | **Skin swab culture** | **Ureteral stent culture** | **mNGS Species Name:Reads number** | **Clinical Opinion Accepted/Unaccepted/Not sure** |
| 1 | S7 | UTI | Male | 57 | - | changes in urine color, character and smell | Providencia stuartii Enterococcus faecalis Morganella morganii | Escherichia coli Proteus vulgaris | | Morganella morganii | Morganella morganii : 3730717 Providencia stuartii : 327924 Enterococcus faecalis : 109982 Pseudomonas aeruginosa : 7197 Human gammaherpesvirus 4 : 3152 | Accepted |
| 2 | S6 | UTI | Female | 77 | - | changes in urine color, character and smell | Klebsiella pneumoniae | - | | Morganella morganii Escherichia coli | Klebsiella pneumoniae : 13397 Pseudomonas aeruginosa : 901 Bacteroides fragilis : 420 Enterococcus faecalis : 286 Morganella morganii : 258 Veillonella parvula : 90 Human betaherpesvirus 5 : 2 | Accepted |
| 3 | S5 | UTI | Male | 74 | - | changes in urine color, character and smell | Escherichia coli Serratia marcescens | Staphylococcus epidermidis Escherichia coli Serratia marcescens | | Corynebacterium mucifaciens Escherichia coli | Veillonella parvula : 383558 Serratia marcescens : 190506 Haemophilus parainfluenzae : 210579 Campylobacter showae : 103107 Streptococcus oralis : 126776 Streptococcus pneumoniae : 2690 Neisseria elongata : 46103 Aggregatibacter aphrophilus : 10822 Acinetobacter baumannii : 5672 Escherichia coli : 1341 Enterococcus faecalis : 2974 Pseudomonas aeruginosa : 105 Human polyomavirus 2 : 1699 | Accepted |
| 4 | S53 | UTI | Male | 68 | - | changes in urine color, character and smell | Staphylococcus aureus Pseudomonas aeruginosa | Klebsiella pneumoniae Streptococcus oralis Staphylococcus aureus | | Staphylococcus aureus Pseudomonas aeruginosa | Pseudomonas aeruginosa : 248545 Staphylococcus aureus : 46850 Enterococcus faecalis : 5702 Streptococcus pneumoniae : 79 Klebsiella pneumoniae : 106 Human gammaherpesvirus 4 : 3 | Accepted |
| 5 | S52 | UTI | Male | 63 | Hypertension | changes in urine color, character and smell | Staphylococcus aureus Klebsiella pneumoniae | Enterococcus faecalis Staphylococcus aureus Klebsiella pneumoniae | | Staphylococcus aureus Klebsiella pneumoniae | Staphylococcus aureus : 244351 Klebsiella pneumoniae : 375884 Enterococcus faecalis : 393 Human gammaherpesvirus 4 : 2 Human betaherpesvirus 6B : 1 | Accepted |
| 6 | S49 | UTI | Male | 72 | - | changes in urine color, character and smell | Proteus vulgaris | Providencia stuartii Staphylococcus aureus Providencia stuartii | | Proteus vulgaris | Proteus penneri : 3044 Proteus vulgaris : 2338 Oligella ureolytica : 1809 Actinomyces urinae : 1767 Enterococcus faecalis : 7 Enterococcus faecium : 6 | Accepted |
| 7 | S48 | UTI | Male | 69 | Diabetes/Hypertension/Coronary heart disease/Erosive gastritis | changes in urine color, character and smell | Proteus vulgaris Morganella morganii Neisseria mucosa Streptococcus oralis | Proteus vulgaris Morganella morganii Staphylococcus aureus | | Proteus vulgaris Morganella morganii Neisseria mucosa | Streptococcus pneumoniae : 929 Morganella morganii : 30555 Achromobacter xylosoxidans : 761 Proteus penneri : 460 Pseudomonas aeruginosa : 27 Enterococcus raffinosus : 121 Enterococcus avium : 109 Citrobacter youngae : 37 Human gammaherpesvirus 4 : 1 Proteus vulgaris : 77 Neisseria mucosa : 2305 Streptococcus oralis : 28082 | Accepted |
| 8 | S46 | UTI | Male | 67 | - | flank pain | Proteus mirabilis Providencia stuartii Proteus vulgaris | Proteus mirabilis Providencia stuartii Staphylococcus aureus | | Proteus mirabilis | Pseudomonas aeruginosa : 261 Providencia stuartii : 7967 Morganella morganii : 1624 Neisseria elongata : 114 Enterococcus faecalis : 46 Human polyomavirus 2 : 5 Proteus mirabilis : 3126 Proteus vulgaris : 799 | Accepted |
| 9 | S39 | UTI | Male | 70 | - | changes in urine color, character and smell | Pseudomonas aeruginosa Neisseria mucosa | - | | Pseudomonas aeruginosa Neisseria mucosa | Pseudomonas aeruginosa : 7119 Streptococcus pneumoniae : 249 Human gammaherpesvirus 4 : 4 Neisseria mucosa : 47492 | Accepted |
| 10 | S38 | UTI | Male | 65 | Hypertension | fever, flank pain | Klebsiella pneumoniae | Klebsiella pneumoniae Enterococcus faecalis | | - | Klebsiella pneumoniae : 20570 Enterococcus faecalis : 42457 Streptococcus anginosus : 6256 Acinetobacter baumannii : 626 Torque teno virus 11 : 36 Human gammaherpesvirus 4 : 2 Human betaherpesvirus 5 : 1 | Accepted |
| 11 | S34 | UTI | Male | - | - | flank pain | Klebsiella pneumoniae Streptococcus oralis Staphylococcus aureus | - | | - | Veillonella parvula : 22363 Streptococcus oralis : 3343 Streptococcus pneumoniae : 68 Haemophilus parainfluenzae : 2160 Neisseria flavescens : 286 Klebsiella quasipneumoniae : 257 Campylobacter showae : 280 Enterococcus faecalis : 352 Staphylococcus epidermidis : 124 Aggregatibacter aphrophilus : 106 Granulicatella adiacens : 98 Finegoldia magna : 83 Staphylococcus aureus : 8 | Accepted |
| 12 | S31 | UTI | Male | 68 | Hypertension | fever, flank pain | Klebsiella pneumoniae Citrobacter freundii | Enterobacter cloacae complex Staphylococcus epidermidis | | Enterobacter cloacae complex Escherichia coli | Klebsiella pneumoniae : 297069 Citrobacter youngae : 26907 Enterobacter cloacae complex : 42507 Escherichia coli : 996 Proteus mirabilis : 12 Pseudomonas aeruginosa : 2 Human polyomavirus 2 : 107 Human gammaherpesvirus 4 : 1 | Unaccepted |
| 13 | S25 | UTI | Female | 73 | - | fever,changes in urine color, character and smell | Proteus mirabilis | - | | Enterococcus faecium | Bacteroides fragilis : 22922 Prevotella copri : 8 Enterococcus faecium : 4 Proteus mirabilis : 2 | Unaccepted |
| 14 | S23 | UTI | Male | 58 | - | changes in urine color, character and smell | Escherichia coli | Escherichia coli Staphylococcus hominis | | Escherichia coli Enterococcus faecalis | Escherichia coli : 5363 | Accepted |
| 15 | S22 | UTI | Female | 68 | Diabetes/Hypertension | changes in urine color, character and smell | Proteus mirabilis | Proteus mirabilis | | Proteus mirabilis | Proteus mirabilis : 1152866 Oligella ureolytica : 1357 | Accepted |
| 16 | S21 | UTI | Male | 58 | - | changes in urine color, character and smell | Providencia rettgeri Alcaligenes faecalis | Providencia stuartii Citrobacter freundii Neisseria macacae | | Providencia stuartii | Providencia rettgeri : 65206 Streptococcus constellatus : 20293 Alcaligenes faecalis : 6905 Citrobacter youngae : 1376 Enterococcus faecalis : 4 Proteus mirabilis : 9 Serratia liquefaciens : 3 | Accepted |
| 17 | S19 | UTI | Female | 82 | Hypertension | changes in urine color, character and smell | Proteus mirabilis Morganella morganii | Staphylococcus aureus Proteus mirabilis Morganella morganii | | Proteus mirabilis Morganella morganii | Proteus mirabilis : 51321 Morganella morganii : 14385 Campylobacter ureolyticus : 8512 Actinotignum schaalii : 6945 Enterococcus faecalis : 6222 Pseudomonas aeruginosa : 88 Escherichia coli : 407 Human gammaherpesvirus 4 : 1 | Accepted |
| 18 | S17 | UTI | Male | 71 | - | changes in urine color, character and smell | Candida auris | Candida auris | | Candida auris | Candida tropicalis : 87377 Human polyomavirus 2 : 8 Human gammaherpesvirus 4 : 1 Human betaherpesvirus 6B : 1 Candida auris : 17 | Accepted |
| 19 | S16 | UTI | Male | 62 | - | changes in urine color, character and smell | Klebsiella pneumoniae | Proteus mirabilis Morganella morganii | | Proteus mirabilis Klebsiella pneumoniae | Streptococcus pneumoniae : 14301 Klebsiella pneumoniae : 145501 Citrobacter youngae : 38854 Proteus mirabilis : 31058 Pseudomonas aeruginosa : 644 Morganella morganii : 16494 | Accepted |
| 20 | S14 | UTI | Male | 61 | Diabetes/Hypertension | flank pain, changes in urine color, character and smell | Escherichia coli | - | | Escherichia coli | Escherichia coli : 1236 Klebsiella pneumoniae : 383 Haemophilus parainfluenzae : 635 Anaerococcus hydrogenalis : 352 Human gammaherpesvirus 4 : 3929 | Accepted |
| 21 | S13 | UTI | Male | 70 | - | changes in urine color, character and smell | Klebsiella pneumoniae | Klebsiella pneumoniae Escherichia coli | | Klebsiella pneumoniae | Klebsiella pneumoniae : 392006 Morganella morganii : 597867 Proteus penneri : 19276 Escherichia coli : 7877 Enterococcus avium : 310 Human polyomavirus 2 : 355 | Accepted |
| 22 | S12 | UTI | Male | 63 | - | changes in urine color, character and smell | Morganella morganii | Escherichia coli | | Morganella morganii | Morganella morganii : 42260 Enterococcus faecalis : 101 Proteus mirabilis : 6 Klebsiella variicola : 3 Anaerococcus lactolyticus : 3 Human polyomavirus 2 : 52 Torque teno virus 24 : 4 Mycoplasma hominis : 1 | Accepted |
| 23 | S11 | UTI | Male | 67 | Hypertension | flank pain | Enterococcus faecalis Klebsiella pneumoniae | staphylococcus warneri Vagococcus fluvialis Klebsiella pneumoniae | | Enterococcus faecium | Klebsiella pneumoniae : 173340 Veillonella parvula : 61251 Enterococcus faecalis : 35291 Prevotella melaninogenica : 350 Human gammaherpesvirus 4 : 2 Human gammaherpesvirus 8 : 1 | Accepted |
| 24 | S10 | UTI | Female | 67 | Hypertension | flank pain, changes in urine color, character and smell | Serratia marcescens Proteus penneri | Klebsiella pneumoniae Providencia stuartii | | Proteus mirabilis | Serratia marcescens : 1099983 Proteus mirabilis : 60276 Morganella morganii : 15658 Oligella ureolytica : 885 Human gammaherpesvirus 4 : 1 Proteus penneri : 104 | Accepted |
| 25 | S51 | ASB | Male | 72 | Hypertension | NO | Klebsiella pneumoniae Staphylococcus aureus | Klebsiella pneumoniae Enterococcus faecalis Escherichia coli | | Klebsiella pneumoniae | Klebsiella pneumoniae : 442459 Staphylococcus aureus : 9461 Streptococcus constellatus : 47736 Pseudomonas aeruginosa : 3787 Enterococcus faecalis : 35687 Oligella ureolytica : 14327 Human polyomavirus 2 : 396 | Accepted |
| 26 | S4 | ASB | Male | 76 | - | NO | Enterococcus faecalis Enterobacter cloacae complex | Enterococcus faecium Staphylococcus haemolyticus Enterobacter cloacae complex | | Enterococcus faecalis Enterobacter cloacae complex | Alcaligenes faecalis : 30987 Enterobacter cloacae complex : 25053 Enterococcus faecalis : 12571 Candida albicans : 1 Human gammaherpesvirus 4 : 1 | Accepted |
| 27 | S44 | ASB | Male | 57 | - | NO | Klebsiella oxytoca | Klebsiella oxytoca Staphylococcus aureus Streptococcus sanguinis Enterococcus faecalis | | Klebsiella oxytoca Staphylococcus aureus | Haemophilus parainfluenzae : 60634 Klebsiella michiganensis : 1306 | Accepted |
| 28 | S43 | ASB | Male | 68 | Hypertension | NO | Enterobacter cloacae complex | Enterobacter cloacae complex | | - | Enterobacter cloacae complex : 2275310 Citrobacter youngae : 241735 Klebsiella pneumoniae : 751 Human polyomavirus 2 : 19 Human polyomavirus 5 : 1 | Accepted |
| 29 | S42 | ASB | Male | 61 | - | NO | Staphylococcus aureus Proteus mirabilis | - | | Staphylococcus aureus Proteus mirabilis | Oligella ureolytica : 34771 Enterococcus faecalis : 15815 Staphylococcus aureus : 1553 Proteus mirabilis : 7110 Morganella morganii : 50 Human polyomavirus 2 : 1 | Accepted |
| 30 | S41 | ASB | Male | 78 | Hypertension | NO | Klebsiella pneumoniae Acinetobacter baumannii | Klebsiella pneumoniae Burkholderia cepacia Streptococcus mitis | | Klebsiella pneumoniae Candida tropicalis Candida albicans | Klebsiella quasipneumoniae : 319847 Enterococcus faecalis : 6099 Torque teno virus 5 : 4 Human polyomavirus 2 : 5 Human gammaherpesvirus 4 : 3 Klebsiella pneumoniae : 39701 Acinetobacter baumannii : 10 | Accepted |
| 31 | S40 | ASB | Male | 63 | - | NO | Enterococcus faecium Staphylococcus haemolyticus | Enterococcus faecium Staphylococcus haemolyticus | | - | Enterococcus faecium : 2786872 Burkholderia multivorans : 192106 Klebsiella pneumoniae : 27 Human gammaherpesvirus 4 : 3 | Unaccepted |
| 32 | S3 | ASB | Male | 67 | - | NO | Enterococcus faecalis Staphylococcus aureus | Enterococcus faecalis Staphylococcus aureus | | Enterococcus faecalis Staphylococcus aureus | Staphylococcus aureus : 410 Haemophilus parainfluenzae : 1626 Enterococcus faecalis : 1182 Actinomyces urogenitalis : 333 Human polyomavirus 2 : 396 Torque teno virus 16 : 3 Human gammaherpesvirus 4 : 2 | Accepted |
| 33 | S36 | ASB | Female | 67 | - | NO | Escherichia coli Staphylococcus haemolyticus Enterococcus faecalis | Enterococcus faecalis Escherichia coli Staphylococcus haemolyticus | | Escherichia coli | Enterococcus faecalis : 979 Veillonella parvula : 800 Escherichia coli : 292 Klebsiella pneumoniae : 19 Bacteroides fragilis : 25 Candida albicans : 3 Staphylococcus haemolyticus : 739 | Accepted |
| 34 | S33 | ASB | Female | 69 | Hypertension | NO | Escherichia coli Enterococcus faecium | Escherichia coli Enterococcus casseliflavus Enterococcus faecium | | Escherichia coli | Escherichia coli : 23960 Enterococcus faecium : 26 | Accepted |
| 35 | S30 | ASB | Male | 77 | - | NO | Klebsiella pneumoniae Streptococcus oralis | Klebsiella pneumoniae Staphylococcus epidermidis Streptococcus oralis | | Staphylococcus epidermidis Streptococcus oralis | Veillonella parvula : 8608 Neisseria flavescens : 479 Streptococcus oralis : 1685 Streptococcus pneumoniae : 43 Campylobacter showae : 641 Enterococcus faecalis : 797 Klebsiella quasipneumoniae : 242 Klebsiella pneumoniae : 33 Aggregatibacter aphrophilus : 54 Granulicatella adiacens : 69 Finegoldia magna : 51 | Accepted |
| 36 | S2 | ASB | Male | 56 | - | NO | Morganella morganii | Morganella morganii Corynebacterium amycolatum | | Morganella morganii Enterococcus faecalis | Morganella morganii : 504068 Human polyomavirus 2 : 6 Human polyomavirus 5 : 2 Torque teno midi virus 7 : 1 | Accepted |
| 37 | S29 | ASB | Male | 59 | Hypertension | NO | Escherichia coli Shigella sonnei | Corynebacterium striatum Enterococcus gallinarum | | Escherichia coli | Escherichia coli : 3222 Enterococcus faecalis : 1814 Bacteroides uniformis : 74 Clostridium butyricum : 129 Human polyomavirus 2 : 64 Torque teno virus 5 : 15 Shigella sonnei : 7138 | Accepted |
| 38 | S28 | ASB | Male | 72 | - | NO | Providencia stuartii Escherichia coli | - | | Providencia stuartii Escherichia coli | Providencia stuartii : 3951305 Enterococcus faecalis : 451929 Escherichia coli : 620 | Accepted |
| 39 | S27 | ASB | Male | 57 | - | NO | Klebsiella oxytoca Morganella morganii Neisseria mucosa | - | | Proteus vulgaris Klebsiella oxytoca | Fusobacterium hwasookii : 8985 Aggregatibacter segnis : 12083 Campylobacter concisus : 9676 Providencia stuartii : 9581 Prevotella melaninogenica : 4215 Porphyromonas asaccharolytica : 2152 Morganella morganii : 3678 Enterococcus faecalis : 1727 Pseudomonas aeruginosa : 143 Streptococcus anginosus : 950 Veillonella parvula : 692 Neisseria mucosa : 197 Finegoldia magna : 735 Acinetobacter baumannii : 281 Klebsiella oxytoca : 337 | Accepted |
| 40 | S26 | ASB | Male | 78 | - | NO | Escherichia coli Klebsiella pneumoniae | Enterococcus faecalis Enterococcus avium | | Escherichia coli Klebsiella pneumoniae Enterococcus faecalis | Klebsiella pneumoniae : 144393 Escherichia coli : 14799 Human polyomavirus 2 : 1865 Torque teno mini virus 18 : 2 Human alphaherpesvirus 1 : 1 | Accepted |
| 41 | S24 | ASB | Male | 72 | - | NO | Proteus mirabilis | Proteus mirabilis | | Proteus mirabilis | Proteus mirabilis : 18849 | Accepted |
| 42 | S20 | ASB | Male | 61 | Hypertension | NO | Escherichia coli | Morganella morganii Staphylococcus haemolyticus Klebsiella pneumoniae | | Klebsiella pneumoniae Escherichia coli | Streptococcus anginosus : 9332 Streptococcus pneumoniae : 132 Bacteroides fragilis : 14654 Pseudomonas aeruginosa : 580 Fenollaria massiliensis : 2423 Veillonella parvula : 2021 Aggregatibacter segnis : 1862 Morganella morganii : 1861 Actinomyces nasicola : 964 Eikenella corrodens : 932 Gemella morbillorum : 616 Peptococcus niger : 648 Peptostreptococcus anaerobius : 564 Escherichia coli : 192 Varibaculum cambriense : 386 Helcococcus sueciensis : 229 Alcaligenes faecalis : 202 Proteus penneri : 61 Enterococcus avium : 25 Torque teno virus 22 : 3 | Accepted |
| 43 | S1 | ASB | Female | 57 | - | NO | Pseudomonas aeruginosa Morganella morganii | Staphylococcus simulans Proteus vulgaris Enterococcus casseliflavus | | Providencia stuartii | Morganella morganii : 8333 Providencia stuartii : 3965 Pseudomonas aeruginosa : 172 Proteus vulgaris : 2158 Haemophilus influenzae : 9 Enterococcus faecalis : 134 Human polyomavirus 2 : 48 | Accepted |
| 44 | S15 | ASB | Male | 62 | - | NO | Serratia marcescens | Staphylococcus epidermidis Serratia marcescens Enterococcus faecalis | | Serratia marcescens | Serratia marcescens : 89336 Enterococcus faecalis : 560 Klebsiella quasipneumoniae : 15 Human polyomavirus 2 : 4 Human gammaherpesvirus 4 : 2 | Accepted |
