# Supplementary material for: Screening Biomarkers and Constructing a Predictive Model for Symptomatic Urinary Tract Infection and Asymptomatic Bacteriuria in Patients Undergoing Cutaneous Ureterostomy: A Metagenomic Next-Generation Sequencing Study
Source: Dis Markers. 2022 Apr 28;2022:7056517. doi: 10.1155/2022/7056517 (PMC9072028; doi:10.1155/2022/7056517)
Supplement: Supplementary Materials — Supplementary Table S1: clinic characteristics of patients included in this study. Supplementary Table S2: data production of 44 samples. [file 7056517.f1.zip › Supplementary Table S2_Data production of 44 samples.doc]

| **Supplementary Table S2 Data production of 44 samples** | | | | | | | | | | | | |
| --- | --- | --- | --- | --- | --- | --- | --- | --- | --- | --- | --- | --- |
| **#Sample** | **RawReads(#)** | **Adapter_ratio(%)** | **Duplication(%)** | **Clean_GC(%)** | **Clean_Q20** | **Clean_Q30** | **LowQuality(%)** | **Nfilter(%)** | **TooShort(%)** | **CleanReads** | **Effective(%)** | **LowComplexity(%)** |
| S6 | 56645033 | 0.21 | 7.99 | 42.14 | 98.67 | 93.9 | 0 | 0 | 0.01 | 51451871 | 90.83 | 1.16 |
| S21 | 33705144 | 0.21 | 7.83 | 41.94 | 98.1 | 91.71 | 0.01 | 0 | 0.01 | 30670734 | 91 | 1.15 |
| S12 | 39967601 | 0.21 | 6.81 | 40.58 | 98.28 | 92.15 | 0 | 0 | 0.01 | 36806901 | 92.09 | 1.09 |
| S27 | 28724666 | 0.22 | 6.17 | 40.8 | 97.63 | 90.61 | 0.01 | 0 | 0.01 | 26596149 | 92.59 | 1.21 |
| S29 | 24021202 | 0.21 | 6.4 | 40.65 | 97.69 | 90.68 | 0.01 | 0 | 0.01 | 22200706 | 92.42 | 1.16 |
| S25 | 50822701 | 0.21 | 6.72 | 42.35 | 98.07 | 92.09 | 0.01 | 0 | 0.01 | 46857454 | 92.2 | 1.06 |
| S19 | 64210418 | 0.21 | 6.3 | 41.88 | 98.35 | 92.72 | 0 | 0 | 0.01 | 59354875 | 92.44 | 1.25 |
| S2 | 34135057 | 0.2 | 7.01 | 41.46 | 97.97 | 91.38 | 0.01 | 0 | 0.01 | 31314613 | 91.74 | 1.23 |
| S48 | 38820542 | 0.21 | 6.2 | 42.03 | 98.3 | 92.49 | 0.01 | 0 | 0.01 | 35907918 | 92.5 | 1.28 |
| S53 | 39050926 | 0.21 | 5.69 | 44.8 | 97.78 | 90.45 | 0.01 | 0 | 0.01 | 36385531 | 93.17 | 1.11 |
| S15 | 42802107 | 0.22 | 6.27 | 42.07 | 98.01 | 91.34 | 0.01 | 0 | 0.01 | 39618419 | 92.56 | 1.14 |
| S49 | 38667875 | 0.21 | 6.92 | 42.98 | 98.77 | 94.11 | 0 | 0 | 0.01 | 35545691 | 91.93 | 1.14 |
| S10 | 37118606 | 0.2 | 6.5 | 43.62 | 98.09 | 91.63 | 0.01 | 0 | 0.01 | 34283271 | 92.36 | 1.12 |
| S33 | 49051357 | 0.2 | 8.19 | 41.71 | 98.31 | 92.77 | 0.01 | 0 | 0.01 | 44294005 | 90.3 | 1.48 |
| S46 | 30402390 | 0.21 | 5.97 | 41.96 | 98.3 | 92.17 | 0 | 0 | 0.01 | 28227205 | 92.85 | 1.17 |
| S52 | 55119952 | 0.21 | 5.24 | 42.19 | 97.97 | 91.34 | 0.01 | 0 | 0.01 | 51596518 | 93.61 | 1.12 |
| S20 | 23857506 | 0.21 | 5.69 | 41.62 | 97.22 | 88.67 | 0.01 | 0 | 0.01 | 22210251 | 93.1 | 1.19 |
| S44 | 52107255 | 0.21 | 6.11 | 42.13 | 98.37 | 92.54 | 0 | 0 | 0.01 | 48264567 | 92.63 | 1.25 |
| S31 | 63496407 | 0.2 | 6.37 | 42.3 | 98.57 | 93.39 | 0 | 0 | 0.01 | 58621785 | 92.32 | 1.29 |
| S36 | 32768029 | 0.21 | 5.78 | 41.58 | 98.46 | 92.83 | 0 | 0 | 0.01 | 30473102 | 93 | 1.2 |
| S17 | 52379891 | 0.21 | 7.23 | 41.93 | 98.39 | 92.99 | 0.01 | 0 | 0.01 | 47734944 | 91.13 | 1.61 |
| S30 | 37613592 | 0.21 | 5.77 | 41.86 | 98.07 | 91.4 | 0 | 0 | 0.01 | 34975779 | 92.99 | 1.22 |
| S34 | 62760045 | 0.21 | 5.54 | 41.62 | 98.29 | 92.25 | 0 | 0 | 0.01 | 58484902 | 93.19 | 1.26 |
| S5 | 78099112 | 0.2 | 8.23 | 43.8 | 98.43 | 93.05 | 0.01 | 0.15 | 0.01 | 70355386 | 90.08 | 1.5 |
| S14 | 58493249 | 0.2 | 9.03 | 43.67 | 98.19 | 92.64 | 0.02 | 0.15 | 0.01 | 52130896 | 89.12 | 1.65 |
| S23 | 43653878 | 0.21 | 6.05 | 42.97 | 98.13 | 91.72 | 0.01 | 0.15 | 0.01 | 40365698 | 92.47 | 1.32 |
| S11 | 78048992 | 0.2 | 7.46 | 42.26 | 98.35 | 92.71 | 0.01 | 0.15 | 0.01 | 70919579 | 90.87 | 1.49 |
| S3 | 65152711 | 0.3 | 6.27 | 41.36 | 98.34 | 92.2 | 0 | 0.14 | 0.01 | 60169475 | 92.35 | 1.22 |
| S4 | 62812245 | 0.5 | 6.81 | 41.95 | 98.49 | 92.8 | 0 | 0.14 | 0.01 | 57574551 | 91.66 | 1.37 |
| S1 | 84098160 | 0.34 | 5.96 | 41.65 | 98.42 | 92.5 | 0 | 0.14 | 0.01 | 77888565 | 92.62 | 1.27 |
| S7 | 56135826 | 4.02 | 14.63 | 46.55 | 98.63 | 93.34 | 0 | 0 | 0.02 | 47395632 | 84.43 | 0.9 |
| S28 | 59138007 | 3.73 | 14.54 | 43.65 | 98.36 | 92.17 | 0 | 0 | 0.02 | 49461447 | 83.64 | 1.78 |
| S24 | 66260919 | 0.49 | 9.4 | 43.38 | 98.24 | 91.8 | 0 | 0 | 0.01 | 58905124 | 88.9 | 1.67 |
| S26 | 55429445 | 0.53 | 8.99 | 41.17 | 98.43 | 92.27 | 0 | 0 | 0.01 | 49644356 | 89.56 | 1.43 |
| S41 | 40052886 | 0.38 | 8.2 | 42.73 | 98.05 | 91.03 | 0 | 0 | 0.01 | 36219460 | 90.43 | 1.35 |
| S42 | 32459328 | 0.9 | 9.43 | 43.65 | 97.77 | 90.05 | 0.01 | 0 | 0.02 | 28981124 | 89.28 | 1.26 |
| S43 | 36186528 | 0.58 | 7.43 | 44.17 | 97.88 | 90.44 | 0 | 0 | 0.01 | 33182178 | 91.7 | 0.85 |
| S39 | 82232447 | 0.44 | 5.84 | 41.75 | 98.3 | 91.95 | 0 | 0 | 0.01 | 76514604 | 93.05 | 1.1 |
| S51 | 68493077 | 0.53 | 6.42 | 41.86 | 97.7 | 89.75 | 0 | 0 | 0.01 | 63018963 | 92.01 | 1.56 |
| S16 | 72558102 | 0.84 | 7.2 | 42.62 | 98.08 | 91.15 | 0 | 0 | 0.01 | 66437668 | 91.56 | 1.22 |
| S38 | 82856417 | 0.41 | 6.18 | 41.89 | 98.24 | 91.66 | 0 | 0 | 0.01 | 76649328 | 92.51 | 1.29 |
| S22 | 54750012 | 0.34 | 5.44 | 41.83 | 98.28 | 91.93 | 0.01 | 0 | 0.01 | 51155667 | 93.43 | 1.1 |
| S13 | 76088511 | 0.32 | 7.99 | 43.46 | 98.66 | 93.63 | 0.01 | 0 | 0.01 | 69033966 | 90.73 | 1.27 |
| S40 | 64124035 | 0.31 | 13.46 | 43.9 | 98.34 | 92.5 | 0 | 0 | 0.01 | 54571075 | 85.1 | 1.42 |
